# Mother–child dyads double burden of malnutrition and associated factors in Burkina Faso

**DOI:** 10.1017/S1368980026102389

**Published:** 2026-03-26

**Authors:** Hermann Biénou Lanou, Aristide Marie Arsène Koumbem, Jérôme Winbetouréfâ Somé, Boubacar Savadogo, Aristide Romaric Bado, Seni Kouanda

**Affiliations:** Institut de Recherche en Sciences de la Santé (IRSS), Centre National de la Recherche Scientifique et Technologique (CNRST), Ouagadougou, Burkina Faso

**Keywords:** Double burden of malnutrition, Overweight, Underweight, Dyad, Burkina Faso

## Abstract

**Objective:**

This study aimed to determine the prevalence of mother–child double burden of malnutrition (DBM) based on anthropometric indices and its associated factors in Burkina Faso.

**Design:**

This cross-sectional study used nationally representative data from the 2021 Burkina Faso Demographic and Health Survey (BFDHS-V). DBM was defined as follows: overweight mother with stunted child (OM/SC); overweight mother with wasted child (OM/WC); overweight mother with underweight child (OM/UC); overweight mother with stunted or wasted or underweight child (OM/SC-WC-UC). Generalised linear model of regression using R programming was performed to identify factors associated with DBM.

**Setting:**

Burkina Faso

**Participants:**

5286 mother–child dyads living in the same household.

**Results:**

The prevalence of DBM in mother–child dyads was 4·9 % for OM/SC-WC-UC. Urban residence was inversely associated with 3 forms of dyads DBM. OM/SC-WC-UC: aOR = 0·60, 95 % CI (0·37, 0·96), OM/WC: aOR = 0·23, 95 % CI (0·11, 0·45), and OM/UC: aOR = 0·51, 95 % CI (0·29, 0·89). Higher child birth order was associated with increased odds of OM/WC: aOR = 3·82, 95 % CI (1·21, 12·10) and OM/UC: aOR = 4·75, 95 % CI (1·65–13·62). Older maternal age was associated with OM/SC: aOR = 3·17, 95 % CI (1·44, 7·00) and belonging to a wealthier household was associated with OM/SC-WC-UC: aOR = 3·43, 95 % CI (1·61, 7·30).

**Conclusions:**

The finding suggests that household-level DBM is an emerging problem in Burkina Faso. The most prevalent form of DBM includes an overweight mother with a stunted child, and common associated factors include urban residence and high socio-economic status. Urgent strategies and actions need to be put in place in order to avert this trend.

Over the past decades, many low- and middle-income countries (LMICs) have undergone significant changes in the nutritional profile of its population, characterised by an increasing prevalence of overweight/obesity associated with the persistence or slight decline in undernutrition.^(1)^ According to the recent estimates, 149·2 and 45·4 million children under 5 years of age suffer from stunting and wasting,^(2)^ respectively, while an estimated 37 million are overweight.^(3)^ Concurrently, 40 % of adult men and women are overweight or obese, and 29 % of adolescent girls and women of childbearing age are affected by anaemia.^(2)^ This situation results in the coexistence of contrasting forms of malnutrition under the term of double burden of malnutrition (DBM). The DBM usually refers to the coexistence of undernutrition and overnutrition, whether within the same individual, the same household or the same community.

The DBM has serious developmental, economic and long-term health consequences for individuals and nations. Poor nutritional status in children under 5 years of age is associated with short- and long-term health complications such as cognitive impairment and cardiometabolic diseases,^(4)^ and nutrition-related factors contribute to approximately 45 % of deaths in this population.^(5)^ Maternal obesity is linked to gestational diabetes, pre-eclampsia, haemorrhage and a higher risk of neonatal and infant death.^(6)^ In addition, obesity leads to impaired economic productivity and increased expenditure on health care.^(7)^ Understanding the forms and correlated factors of DBM provides an opportunity to identify the drivers, which is critical for designing and targeting interventions to improve the health and well-being of both mothers and children as well as achieving the second sustainable development goal.

At national level, the DBM was reported in 38 % of countries in 2010, based on a definition that either wasting or thinness in women with overweight among adults or children.^(1)^ This form of malnutrition is particularly prevalent in sub-Saharan Africa (SSA), South Asia, East Asia and the Pacific.^(1)^ Social groups such as the urban poor, the rural rich and people living in slum conditions have been found to be the most affected by DBM.^(8,9)^ DBM at the household level is characterised by the co-occurrence of overnutrition and undernutrition within the same household in at least two people. Although there is no single operational definition or indicator of the DBM at the household level,^(10)^ the most common association is undernourished child and overweight or obese mother, commonly referred to as mother–child DBM. Studies on household-level DBM with couples of overweight mothers and undernourished children have reported a prevalence ranging from 5 % in sub-Saharan African countries^(11)^ to 56 % in South-East Asia.^(12)^ However, most studies used different cut-off points of anthropometric indicators to define undernutrition and overnutrition. Such heterogeneity in DBM definitions and indicators has consequently resulted in a wide range of prevalence estimates of household-level DBM between studies.^(10)^

Household-level DBM is often considered paradoxical, as the two forms of malnutrition were understood to arise from two different sets of determinants,^(12)^ and addressing this phenomenon would therefore be challenging. Also, because the members of these families usually shared resources, eating behaviours and household micro-environment,^(13)^ an alike nutritional outcome would be expected from household’s members who share the same foods with comparable dietary intake and pattern at the household level. Therefore, the two forms should not be considered distinct conditions at opposite tails of the nutrition spectrum, but rather indicative of malnutrition including low-quality diets, high in calories but low in proteins and micronutrients.^(14)^

The key driving mechanism of household-level DBM often relates to the nutrition transition and related changes in household dietary and lifestyle patterns.^(15)^ The underlying factors are sedentary lifestyle, lack of dietary diversity, poor infant and child feeding and care practices, poor water and sanitation, all of which leading to poor dietary intake (quality and quantity), physical inactivity and disease.^(16)^ It is also recognised that a combination of maternal overweight/obesity and child undernutrition is the result of an interaction of factors such as the socio-economic status of the household, dietary habits and intensity of physical activities.^(1,15)^

Burkina Faso is one of the SSA country where nutrition transition appears to be accelerating with an increasing prevalence of overweight and obesity mainly among the urban women,^(17)^ and a shift in the diet profile^(18)^ and cardiometabolic risk factors.^(19)^ Despite progress made in various sectors in the country in relation to wasting reduction, there is persistence of underweight and stunting among children and a rising overweight and obesity among women and children especially in urban households. Although the DBM is an increasing public health concern, some evidence indicates that its occurrence at the household level may be largely random.^(20)^ Preliminary analysis revealed that the co-occurrence of maternal overweight with child stunting, wasting or underweight is less frequent than would be expected if these conditions were independent. This suggests dissociative underlying risk factors at the population level. Consequently, households that do experience these specific DBM forms represent a critical sub-population where unique contextual factors may override this dissociative trend. Therefore, the objective of this study is to identify the specific socio-economic, demographic and environmental predictors that characterise households experiencing these distinct DBM forms, providing insights for targeted interventions.

## Material and methods

### Data source and survey design

**Figure 1.**
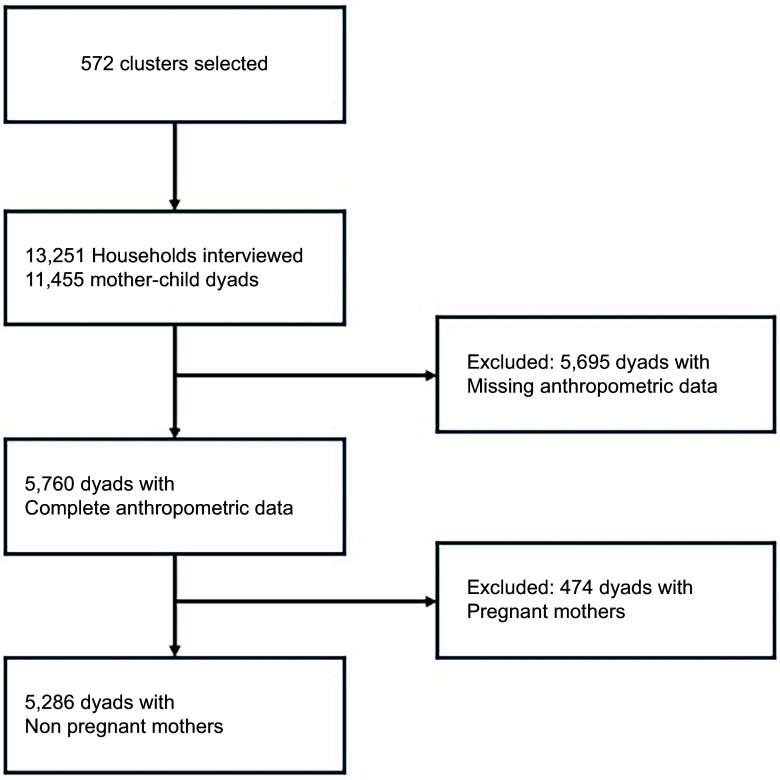
Flowchart describing the sample selection procedure for the analysis of mother–child dyads’ double burden of malnutrition in Burkina Faso.

This study is a secondary analysis of data from the 2021 Burkina Faso Demographic and Health Survey (BFDHS-V). BFDHS-V was a nationally representative cross-sectional survey conducted between 30 July and 30 November 2021 by the National Bureau of Statistics of Burkina Faso and The Demographic and Health Survey Program ICF (Rockville, Maryland, USA). It employed a two-stage, stratified area sampling design, and the sample size was calculated to provide representative estimates at the national level, for the capital city (Ouagadougou), for urban areas other than Ouagadougou and for rural areas. The survey selected 13 251 households (98 % response rate). A total of 17 659 women of reproductive age (15–49 years) were interviewed, including the completion of full household questionnaires and anthropometric measurements for children < 5 years and for the women themselves. For this study, a weighted subsample of 5760 mother−child dyads with complete anthropometric data was considered. After excluding 474 women due to pregnancy, a final sample of 5286 dyads was retained for analysis (Figure 1).

### Outcome variables

#### Definitions

The outcome variable was the household-level DBM, defined as a mother and her child living in the same household, each simultaneously presenting at least one form of malnutrition. Maternal malnutrition was defined as the presence of overweight/obesity, while child’s malnutrition was defined as stunting, wasting or underweight. Accordingly, household-level DBM was classified into the following four forms: maternal overweight with child stunting (OM/SC); maternal overweight with child wasting (OM/WC); maternal overweight with child underweight (OM/UC); and maternal overweight with child stunting, wasting or underweight (OM/SC-WC-UC).

#### Anthropometric measurements and nutritional status

For the BFDHS-V, weight measurements were taken using digital scales (SECA® 878U). Height/length measurements were taken using a height board (ShorrBoard®). Children under 24 months of age were measured in the supine position, while older children and adults were measured in the standing position. Child nutritional status was determined using the 2006 WHO growth standard to calculate z-scores. Children were considered undernourished if they were stunted, wasted or underweight, defined as height-for-age, weight-for-age and weight-for-height/length z-scores below –2 sd, respectively. Maternal nutritional status was classified based on BMI as follows: underweight (<18·5 kg/m^2^), normal weight (18·5–24·9 kg/m^2^), overweight (25·0–29·9 kg/m^2^) and obese (≥30·0 kg/m^2^).

### Explanatory variables

The explanatory variables included were identified from previous literature.^(15,21)^ The variables are composed of individual characteristics of children and their parents (child’s age, sex, birth order, size at birth, breastfeeding history; mother’s age, parent’s educational and working status) and those related to the household as follows: household size, place of residence, toilet facilities and wealth index. Mothers’ age was categorised into four groups: ‘<25 years’, ‘25–29 years’, ‘30–34 years’ and ‘≥ 35 years’. The wealth index was a composite score calculated using principal component analysis and provided in the BFDHS-V dataset. The index was based on the household’s ownership of selected assets (television, refrigerator, bicycle, animals, agricultural land, gardens, etc.), materials used for housing construction, and types of water access and sanitation facilities.

### Statistical analysis

For descriptive statistics, we reported weighted frequency distribution to summarise the categorical variables and means (standard errors) for the continuous variables. Owing to the two-stage stratified cluster sampling design of the BFDHS-V, the recommended multilevel modelling for Demographic and Health Survey data was used in this analysis.^(22)^ Prior to regression modelling, we assessed whether the observed co-occurrence of maternal overweight and child undernutrition represented meaningful patterning rather than random association. We calculated the expected prevalence of each DBM form under the assumption of statistical independence between maternal overweight and child undernutrition indicators. Expected prevalence was computed as prevalence of maternal overweight (P_maternal overweight_) * prevalence of child undernutrition (P_child undernutrition_) * 100. We compared observed and expected prevalence using chi-square tests. The association between the outcomes and the household, parent and child characteristics was assessed using a generalised linear model for complex survey data.^(23)^ The *‘svyglm’* (survey generalised linear model) regressions were performed using the ‘survey’ package in R program. (version 4·4·2). Statistical significance was set at *P < 0·05*. All significant variables and non-significant variables (at *P < 0·20*) in the bivariate analysis were included in the multivariable analysis to determine their collective associations with DBM. Multicollinearity between the independent variables was tested using the variance inflation factor with a cut-off point of variance inflation factor = 3, ensuring that the independent variables were not highly correlated. Associations were expressed as adjusted OR (aOR) with 95 % CI.

## Results

### Characteristics of the study sample

Table 1 describes the household and sociodemographic characteristics of the participants. The households in the study had about eight members on average, and 74·7 % of them were located in rural areas. The mean maternal age was 29·6 (sd = 7·02) years. Of all mothers, 70·3 % had no formal education, while only 4·3 % attained higher education. The mean maternal BMI was 22·6 (4·1) kg/m^2^. Among mothers, the prevalence of overweight/obesity and underweight was 20·9 % and 7·9 %. Overall, 21·9 % of the children were stunted and 10·8 % were wasted. Only 1·3 % and 1·0 % of children were overweight and obese, respectively (Table 1).

**Table 1.** Sociodemographic characteristics of the participants

Characteristics	*N*	%
Household size		
≤4	798	15·3
5 or 6	1240	24·0
≥7	3248	60·7
Number of children < 5	2·27	0·04
1	1595	30·5
2 or 3	2946	55·3
4 or 5	745	14·2
Place of residence		
Urban	1325	25·3
Rural	3780	74·7
Wealth quintile		
Poorest	1001	20·67
Poorer	1039	20·25
Middle	1216	21·62
Richer	1137	19·61
Richest	893	17·86
Mother’s age	29·7	0·14
<25 years	1419	26·4
25–29 years	1313	25·4
30–34 years	1123	21·4
≥35 years	1401	26·8
Mother’s nutritional status	22·6	0·09
BMI < 18·5 kg/m^2^	369	7·9
18·5 ≤ BMI < 25·0 kg/m^2^	3770	71·2
25·0 ≤ BMI < 30·0 kg/m^2^	862	15·3
BMI ≥ 30 kg/m^2^	285	5·6
Mother’s education		
No education	3694	70·3
Primary education	682	12·5
Secondary education	675	12·9
Higher education	235	4·3
Mother’s working status		
Not working	1753	34·5
Worked in the past	214	3·8
Currently working	3319	61·7
Father’s education		
No education	3260	74·3
Primary	606	11·9
Secondary	543	11·2
Higher	130	2·7
Father’s work		
Do not work	484	10·2
Technical/professional/clerical	347	7·2
Sales	763	15·4
Agriculture/self-employed	2299	45·7
Household/domestic/manual	1088	21·4
Child’s sex		
Female	2599	49·1
Child’s birth order		
1	1113	21·3
2 or 3	1857	35·1
4 or 5	2316	43·5
Child’s size at birth^†^		
Average	1907	57·9
Smaller than average	265	8·8
Larger than average	1063	33·3
BMI, BMI		

† As perceived by the mother.

### *Preliminary analysis: observed v. expected* double burden of malnutrition *prevalence*

All DBM forms showed significant dissociation, occurring less frequently than expected under the assumption of independence (Table 2). The DBM (OM/SC–WC–UC) indicator was observed in 4.9 % of households, which was significantly lower than the 6.7 % expected by chance (*P* = 0.023). Similar dissociative patterns were observed for the OM/SC and OM/UC combinations, with differences reaching up to 1.5 percentage points (all *P* < 0.05).

**Table 2.** Comparison of observed and expected prevalence of DBM forms

DBM form	Observed %	95 % CI	Expected %^†^	Difference	*P*-value
OM/SC	3·08	2·5–3.7	4·6	−1·5	<0·001
OM/WC	1·8	1·4–2.3	2·3	−0·4	<0·001
OM/UC	2·1	1·7–2.6	3·6	−1·5	0·023
OM/SC-WC-UC	4·9	4·2–5.7	6·7	−1·8	0·023

OM/SC-WC-UC, maternal overweight with child stunting or wasting or underweight. OM/SC, maternal overweight with child stunting. OM/WC, maternal overweight with child wasting. OM/UC, maternal overweight with child underweight.

† Calculated as the product of the prevalence of maternal overweight and the respective child undernutrition indicator x 100.

### Prevalence of double burden of malnutrition

**Figure 2.**
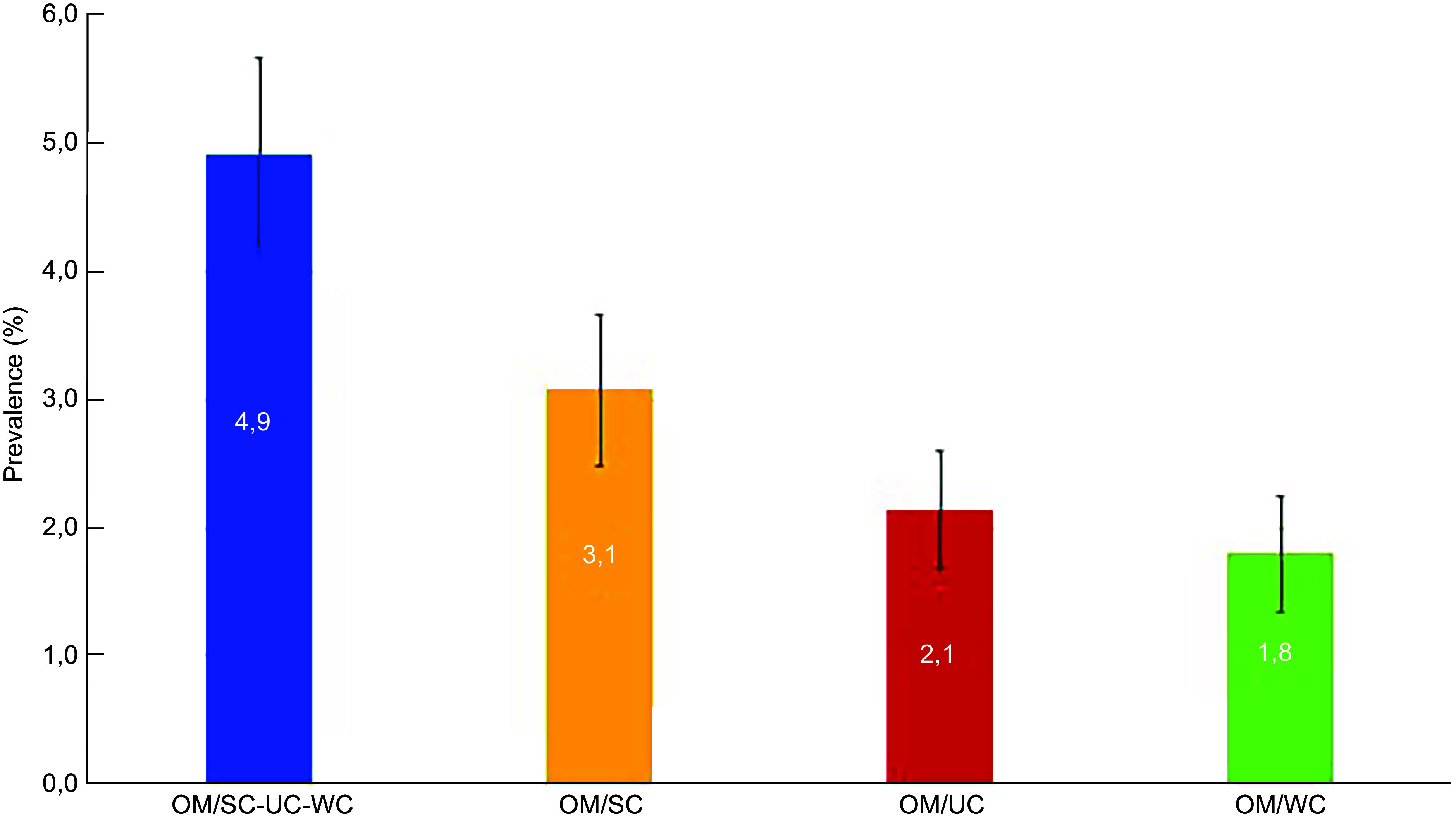
Prevalence (%) of different forms of mother–child dyads’ double burden of malnutrition OM/SC-WC-UC, maternal overweight with child stunting or wasting or underweight. OM/SC, maternal overweight with child stunting. OM/WC, maternal overweight with child wasting. OM/UC, maternal overweight with child underweight.

The prevalence of the different forms of household-level DBM is presented in Figure 1. The highest prevalence (95 % CI) was observed for the composite (OM/SC–WC–UC) form at 4·9 % (4·2–5·7). Lower prevalence estimates were found for the DBM forms combining maternal overweight with each individual type of child undernutrition (Figure 2).

### Factors associated with the double burden of malnutrition

Bivariate analysis showed that the wealth index was significantly associated with all DBM forms. Maternal age and child sex were associated with the OM/SC form, while the household residence, toilet facility status and both maternal and paternal education were associated with the OM/WC and OM/UC forms. The composite form of DBM (OM/SC–WC–UC) was associated with all factors examined except mode of delivery, breastfeeding history and paternal education (Table 3).

**Table 3.** Bivariate logistic regression of predictors of mother–child dyads’ double burden of malnutrition in Burkina Faso

		OM/SC-WC-UC				OM/SC				OM/WC				OM/UC			
Variable	Category	OR	95 %CI		*P*	OR	95 %CI		*P*	OR	95 %CI		*P*	OR	95 %CI		*P*
Residence	Urban	Ref.															
	Rural	0·46	0·33	0·65	***	0·66	0·43	1·01		0·26	0·16	0·42	***	0·39	0·25	0·61	***
Household size	Small (>5)	Ref.															
	Medium (5–6)	1·1	0·75	1·90		0·89	0·50	1·59		1·64	0·82	3·25		1·08	0·58	2·01	
	Large (≥7)	0·83	0·55	1·26		0·84	0·52	1·37		0·76	0·37	1·57		0·63	0·36	1·10	
Wealth index	Poorest	Ref.															
	Poorer	0·72	0·39	1·34		0·86	0·43	1·72		0·59	0·2	1·73		0·39	0·16	0·96	*
	Middle	1·16	0·69	1·94		1·23	0·64	2·35		1·02	0·47	2·2		1·18	0·57	2·46	
	Richer	1·59	0·99	2·55		1·38	0·75	2·51		1·8	0·87	3·73		1·73	0·88	3·43	
	Richest	2·83	1·76	4·56	***	2·49	1·33	4·66	**	3·66	1·88	7·12	***	2·62	1·36	5·06	**
Number of children	1	Ref.															
	2 or 3	0·72	0·52	0·99	*	0·74	0·50	1·09		0·59	0·35	1·01		0·67	0·42	1·05	
	≥4	0·56	0·33	0·95	*	0·63	0·33	1·20		0·57	0·28	1·13		0·55	0·28	1·10	
Drinking water source	Safe	Ref.															
	Unsafe	0·65	0·43	0·99	*	0·70	0·41	1·19		0·39	0·17	0·89	*	0·65	0·35	1·22	
Toilet	Improved	Ref.															
	Not Improved	0·64	0·46	0·87	**	0·70	0·48	1·03		0·60	0·36	0·98	*	0·60	0·37	0·95	*
Mother’s age	<25 y	Ref.															
	25–29 y	1·67	1·03	2·73	*	1·49	0·79	2·72		2·46	1·24	4·89	*	1·17	0·61	2·22	
	30–34 y	1·7	1·03	2·81	*	1·45	0·79	2·67		2·34	1·09	5·03	*	1·25	0·6	2·60	
	≥35 y	2·17	1·38	3·41	***	2·18	1·24	3·82	**	1·94	0·96	3·88		1·36	0·72	2·55	
Mother’s age at first birth	> 18 years	Ref.															
	18–24 years	0·88	0·62	1·24		0·97	0·63	1·48		0·63	0·35	1·14		1·28	0·76	2·14	
	≥25	0·99	0·61	1·61		1·17	0·65	2·11		1·07	0·49	2·36		1·09	0·45	2·65	
Mother’s education	No education	Ref.															
	Primary	1·15	0·68	1·95		0·66	0·35	1·25		2·07	1·04	4·11	*	1·70	0·92	3·13	
	Secondary and higher	1·54	1·07	2·21	*	1·49	0·96	2·29		1·55	0·87	2·75		2·34	1·4	3·91	**
Child birth order	1	Ref.															
	2 or 3	1·46	0·93	2·31		1·27	0·72	2·24		1·85	0·95	3·62		1·83	1·03	3·25	*
	≥4	1·66	1·05	2·62	*	1·39	0·79	2·45		2·00	0·98	4·05		1·35	0·73	2·49	
Mother working status	Never worked	Ref.															
	W. past year	1·68	0·87	3·23		0·87	0·35	2·20		3·37	1·31	8·65	*	0·51	0·12	2·26	
	Currently working	1·13	0·81	1·59		1·15	0·78	1·71		1·48	0·87	2·50		1·15	0·70	1·89	
Father’s work	Don’t work	Ref.															
	Technical & Clerical	1·04	0·53	2·05		1·41	0·54	3·63		0·50	0·17	1·51		1·22	0·41	3·64	
	Sales & services	1·38	0·78	2·45		2·06	0·96	4·41		1·03	0·46	2·34		2·10	0·92	4·8	
	Agriculture/self-employed	0·61	0·37	0·99	*	1·12	0·57	2·18		0·21	0·09	0·49	***	0·59	0·26	1·32	
	Manual, household^†^ domestic	1·09	0·61	1·93		1·41	0·66	3·00		0·88	0·37	2·08		0·98	0·38	2·55	
Child’s sex	Male	Ref.															
	Female	0·57	0·57	0·99	*	0·69	0·49	0·98	*	0·62	0·38	1·01		0·75	0·49	1·14	
Breastfeeding history	No	Ref.															
	Ever/still breastfed	0·84	0·48	1·45		0·72	0·35	1·48		1·28	0·47	3·48		0·68	0·32	1·46	
Father’s education	No education	Ref.															
	Primary	1·1	0·71	1·71		1·10	0·63	1·92		1·25	0·61	2·56		1·39	0·72	2·69	
	Secondary	1·54	0·91	2·61		0·74	0·35	1·58		2·37	1·21	4·63	*	2·10	1·07	4·11	*
	Higher	0·99	0·27	3·04		1·04	0·24	4·43		1·68	0·38	7·34		0·85	0·11	6·43	
Delivery mode	Normal																
	C-section	1·16	0·65	2·40		1·68	0·38	1·03		1·30	0·56	3·03		1·79	0·66	4·83	
Child’s size at birth^‡^	Average	Ref.															
	Smaller	0·96	0·45	2·07		0·48	0·12	1·87		1·08	0·4	2·98		1·48	0·59	3·74	
	Larger	0·66	0·45	0·97	*	0·62	0·38	1·03		0·67	0·38	1·17		0·43	0·22	0·85	*

OM/SC-WC-UC, maternal overweight with child stunting or wasting or underweight. OM/SC, maternal overweight with child stunting. OM/WC, maternal overweight with child wasting. OM/UC, maternal overweight with child underweight. DBM, double burden of malnutrition. P, *P*-value. *P <.05, **P <.01, ***P <.001.

† Includes ‘Domestic work’.

‡ As perceived by the mother.

After adjusting for covariates (Table 3), dyads living in rural areas had significantly lower odds of the three forms of DBM compared to those in urban areas : aOR = 0·23, 95 % CI (0·11, 0·45) for OM/WC ; aOR = 0·51, 95 % CI (0·29, 0·89) OM/UC ; and aOR = 0·60, 95 % CI (0·37, 0·96) for OM/SC-WC-UC (Table 4).

**Table 4.** Multivariable logistic regression to determine the factors associated with mother–child dyads’ double burden of malnutrition in Burkina Faso

		OM/SC-WC-UC				OM/SC				OM/WC				OM/UC			
Variable	Category	aOR	95 %CI		*P*	aOR	95 %CI		*P*	aOR	95 %CI		*P*	aOR	95 %CI		*P*
Residence	Urban	Ref.															
	Rural	0·60	0·37	0·96	*	0·65	0·34	1·27		0·23	0·11	0·45	***	0·51	0·29	0·89	*
Household size	Small (>5)	Ref.															
	Medium (5–6)									1·31	0·60	2·88		0·80	0·36	1·76	
	Large (≥7)									0·83	0·35	1·93		0·43	0·21	0·87	*
Wealth index	Poorest	Ref.															
	Poorer	1·07	0·51	2·26													
	Middle	1·77	0·91	3·45													
	Richer	1·98	1·00	3·90													
	Richest	3·43	1·61	7·30	**												
Number of children	1	Ref.															
	2 or 3	0·86	0·56	1·31		0·87	0·51	1·47		0·56	0·27	1·16		0·56	0·29	1·08	
	≥4	0·74	0·40	1·38		0·52	0·22	1·24		1·00	0·40	2·48		0·73	0·27	1·95	
Drinking water source	Safe	Ref.															
	Unsafe	1·36	0·82	2·25		1·17	0·59	2·32		0·82	0·35	1·92		1·28	0·57	2·88	
Toilet	Improved	Ref.															
	Not Improved	1·22	0·74	1·99		0·88	0·53	1·45		1·18	0·66	2·14		0·68	0·36	1·29	
Mother’s age	<25 y	Ref.															
	25–29 y					2·05	0·86	4·93		1·89	0·79	4·52					
	30–34 y					1·80	0·77	4·24		1·29	0·45	3·71					
	≥35 y					3·17	1·44	7·00	**	0·73	0·21	2·49					
Mother’s age at first birth	> 18 years	Ref.															
	18–24 years	0·87	0·50	1·52													
	≥25	0·84	0·50	1·40													
Mother’s education	No education	Ref.															
	Primary	2·70	1·23	5·92	*	0·82	0·38	1·78		1·81	0·89	3·67		1·92	0·84	4·39	
	Secondary and higher	1·32	0·88	1·97		1·61	0·79	3·28		1·10	0·50	2·39		2·28	0·85	6·10	
Child birth order	1	Ref.															
	2 or 3									1·94	0·75	5·04		4·14	1·58	10·87	**
	≥4									3·82	1·21	12·10	*	4·75	1·65	13·62	**
Mother working status	Never worked	Ref.															
	W. past year									4·76	1·77	12·77	**				
	Currently working									1·30	0·73	2·33					
Father’s work	Don’t work																
	Technical & Clerical																
	Sales & services																
	Agriculture/self-employed																
	Manual, household^†^ domestic																
Child’s sex	Male	Ref.															
	Female	0·74	0·52	1·07		0·71	0·45	1·10		0·58	0·32	1·04		0·66	0·37	1·19	
Breastfeeding history	No																
	Ever/still breastfed																
Father’s education	No education																
	Primary													0·83	0·32	2·11	
	Secondary													0·68	0·25	1·85	
	Higher													0·39	0·04	4·11	
Delivery mode	Normal	Ref.															
	C-section					1·40	0·58	3·38									
Child’s size at birth^‡^	Average	Ref.															
	Smaller	1·13	0·52	2·43		0·51	0·13	1·93		1·22	0·45	3·34		1·17	0·45	3·07	
	Larger	0·63	0·43	0·94	*	0·61	0·36	1·02		0·58	0·32	1·04		0·38	0·19	0·76	**
*VIF*		1·72				2·74				2·24				1·99			

OM/SC-WC-UC, maternal overweight with child stunting or wasting or underweight. OM/SC, maternal overweight with child stunting. OM/WC, maternal overweight with child wasting. OM/UC, maternal overweight with child underweight. aOR, adjusted OR. VIF, variance inflation factor. *P*, p-value. *P <.05, **P <.01, ***P <.001.

† Includes ‘Domestic work’.

‡ As perceived by the mother.

For the OM/WC form of DBM, dyads in which the mother had worked in the past year had higher odds of DBM (aOR = 4·76, 95 % CI (1·77, 12·77)) than those in which the mother had never worked. Similarly, dyads with children of higher birth order (≥4) had greater odds of DBM (aOR = 3·82, 95 % CI (1·61, 12·10)) compared with dyads in which the child had a lower birth order. The odds of the OM/UC form of DBM were lower among dyads living in larger households (≥7 members: aOR = 0·43, 95 % CI (0·21, 0·87)) compared with those living in smaller households. Conversely, this DBM form was more likely among dyads with children of higher birth order (aOR = 4·75, 95 % CI (1·65, 13·62)) compared with those with lower birth order. For the OM/SC–WC–UC form of DBM, dyads in the richest wealth quintile had significantly higher odds (aOR = 3·43, 95 % CI (1·61, 7·30)) compared with those in the poorest quintile. In addition, dyads in which the mother had primary education exhibited increased odds of this DBM form (aOR = 2·70, 95 % CI (1·23,5·92)) compared with those in which the mother had no formal education (Table 4).

## Discussion

In this study, we investigated the coexistence of overnutrition and undernutrition among mother–child dyads in Burkina Faso. Our findings revealed a low prevalence of DBM (4·9 %) within these dyads. Previous evidence from 126 LMIC has reported household-level DBM prevalence ranging from 3 % to 35 %.^(1)^ In Burkina Faso, an earlier estimate based on the previous BFDHS-III data indicated a prevalence of 0·7 %. These findings indicate that the occurrence of DBM has increased nearly fourfold over the past two decades. Despite significant progress in reducing childhood undernutrition in recent years, the prevalence of stunting in children remained above the WHO threshold for public health concern.^(24)^ The most recent National Nutrition Survey indicates that childhood nutritional status has significantly improved over the past decade. In contrast, an increase in overweight prevalence among women of reproductive age has been observed, rising from 9·3 % to 25·3 % over the last two decades.^(24,25)^ This trend calls for urgent initiatives from policymakers and public health authorities to tackle this emerging public health challenge in Burkina Faso.

The three distinct forms of DBM among mother–child dyads–OM/SC, OM/WC, OM/UC–had prevalences of 3·1 %, 2·1 % and 1·8 % respectively, with OM/SC being the most common. A similar pattern has been described in SSA,^(26,27)^ South and Southeast Asia,^(28)^ as well as in Latin America and Caribbean,^(29)^ which may reflect the overall malnutrition profile among children under 5 years, where stunting represents the largest burden and wasting the lowest. Stunting is the indicator of child undernutrition most frequently linked to maternal overnutrition in DBM studies. In SSA, prevalence ranging from 2 % to 6 % was reported in most countries, with the exception of Ghana, where it reached 12 %.^(26)^ The prevalence in our study is lower than that reported in Egypt (12 %) and Guatemala (23 %).^(26)^ When considering any form of child undernutrition coexisting with maternal overweight/obesity, the prevalence of DBM was 4·9 %. This is lower than estimates reported elsewhere: 7·0 % in India,^(30)^ 12·3 % in Ethiopia,^(31)^ 13·5 % in Bangladesh^(32)^ and 12·0 in Southeast Asia.^(28)^ The variations across countries and world regions could be attributed to the level of economic development of these populations and the ongoing process of nutrition transition in these settings. The DBM is commonly associated with nutrition transition characterized by shifts from traditional, nutrient‑dense diets toward greater consumption of ultra‑processed, energy‑dense foods alongside reductions in physical activity driven by urbanization.^(33,34)^ In recent decades, poor economy countries have experienced socio-economic changes and epidemiological transition leading to changes in dietary habits and consumption of energy-dense foods which, along with less physical activity, resulting in a rise of overweight and obesity among adults including mothers.^(35,36)^ Additionally, the diet profile coupled with inadequate water, sanitation and hygiene conditions plays a key role in suboptimal nutrition outcomes in children.^(14)^

We found that several risk factors were associated with an increased likelihood of household DBM. The association between area of residence and DBM in mother–child dyads observed in our study is consistent with the results of an analysis of data from thirty SSA countries, where the odds of an overweight woman and a stunted child co-occurring were found to be higher in urban and peri-urban areas than in rural areas.^(37)^ In contrast, a review of DBM-associated factors found heterogeneous results regarding the place of residence. While some studies identified urban or rural as a risk factor, others found no difference between settings.^(15)^ Furthermore, an analysis of forty-two developing countries revealed that OM/SC was associated with urban residency in Latin America but with rural residency in Africa and Asia,^(38)^ indicating that direction of this association varies by country income level.^(39)^

Consistent with previous research in South and Southeast Asia,^(28)^ Kenya^(27)^ and in Tanzania,^(40)^ we found a positive association between DBM and wealth index. Mother–child dyads in the richest wealth category had higher odds of DBM compared with those in poorer categories. Further, Demographic and Health Survey data from 55 LMIC indicated that DBM is distributed across wealth quintiles, with a higher probability in the richest households of lower-income LMIC and in the poorest households of higher-income LMIC.^(39)^ One possible explanation is that households in the highest wealth index category may experience a dietary transition,^(41)^ characterised by increased consumption of energy-dense nutrient-poor foods and reduced physical activity factors that contribute to a higher risk of overweight and obesity among individuals in these households.^(30,42)^

We found that increasing maternal age was associated with a higher likelihood of dyads experiencing OM/SC form of DBM. This result is consistent with several studies that reported a higher prevalence of DBM among older mothers compared to younger age groups.^(31,43,44)^ The higher likelihood of overweight and obesity among older women in our context^(45)^ and in SSA^(46,47)^ has been well-documented and is likely a key driver of the increased risk of household DBM.

Dyads with children of higher birth order were at higher risk of the OM/WC and OM/UC forms of DBM. This aligns with other studies conducted in Ethiopia^(31)^ and SSA.^(21)^ Higher birth order may reflect a cumulative burden on the household or maternal resources, which can negatively impact the nutritional status of subsequent children. Similarly, the association of DBM and larger household size could be explained by limited access to nutritious food such as fruits, vegetables, meat and fish which are often more expensive than staple foods, potentially contributing to undernutrition among children.^(48)^

Our preliminary results indicated that all DBM forms occurred significantly less frequently than would be expected if maternal overweight and child undernutrition were independent conditions. This suggests that the individuals and socio-economic factors driving maternal overweight and child undernutrition may be mutually protective or operate through distinct pathways in this population. For example, households in extreme poverty may be at high risk for child undernutrition but have low access to energy-dense foods that drive maternal overweight, while more affluent households may have better child nutrition outcomes despite higher maternal BMI. The factors associated with DBM forms are therefore not merely correlates of the individual conditions but may represent a specific convergence of risks that enables this co-occurrence in a distinct group of households.

This study has some limitations. First, because the study design was cross-sectional, we could not establish causal relationships between DBM and the associated factors examined. Second, the BFDHS-V was not specifically designed to investigate DBM; therefore, our analysis was limited to the variables available in the dataset, which may not fully capture the complex interplay of social, community and personal factors influencing DBM. Despite these limitations, this study has several strengths. The use of nationally representative data allowed us to examine, for the first time at the national level, the DBM within mother–child dyads. Additionally, the high response rate enhances the generalisability of our findings and ensures adequate statistical power for the study.

## Conclusion

The findings of this study indicate that maternal overnutrition and child undernutrition co-exist at the household level in Burkina Faso, particularly within mother–child dyads. The occurrence of this DBM was significantly associated with higher household socio-economic status and urban residence. Consequently, mother–child dyads most at risk of DBM were those living in urban areas and belonging to wealthier households. These findings suggest that the DBM is increasingly affecting more advantaged urban populations, where higher socio-economic status elevates the risk of maternal overweight, while child undernutrition persists due to factors such as unequal food distribution, suboptimal child feeding practices and urban lifestyle constraints. Together, these results highlight the need for targeted, double-duty interventions to simultaneously address both undernutrition and overnutrition among these vulnerable groups.
